# Optimization of Ultrasound-Assisted Extraction and Encapsulation of Antioxidants from Orange Peels in Alginate-Chitosan Microparticles

**DOI:** 10.3390/antiox11020297

**Published:** 2022-01-31

**Authors:** Ivan M. Savic, Ivana M. Savic Gajic, Miljana G. Milovanovic, Stanko Zerajic, Dragoljub G. Gajic

**Affiliations:** 1 Faculty of Technology in Leskovac, University of Nis, Bulevar Oslobodjenja 124, 16000 Leskovac, Serbia or vana.savic@yahoo.com (I.M.S.G.); or miljanamilovanovic136@gmail.com (M.G.M.); zerajic@tf.ni.ac.rs (S.Z.); 2 Gajic Associates, Doktora Pantica 77, 14000 Valjevo, Serbia; drago@gajicassociates.com

**Keywords:** orange peel, ultrasound-assisted extraction, optimization, controlled release, encapsulation

## Abstract

The recovery of bioactive compounds from waste and modification of their properties by encapsulation are the main challenges today. In this study, the ultrasound-assisted extraction of antioxidants from orange peels was optimized using a central composite design. Ethanol (50%, *v*/*v*) was the solvent of choice for their extraction. The obtained total antioxidant contents were fitted using the second-order polynomial equation. The optimal conditions were the extraction time of 30 min, temperature of 60 °C, and the liquid-to-solid ratio of 15 mL/g. After that, the optimal extract was encapsulated in alginate-chitosan beads to modify the release of antioxidants under gastrointestinal tract conditions. The average size of beads was 252 µm, while the encapsulation efficiency was 89.2%. The results of the FTIR analysis indicated that there are no interactions between compounds of the extract and alginate-chitosan. In vitro release studies showed an initial rapid and then slow release of antioxidants. This release followed the simple Fickian diffusion. The encapsulation of orange peel extract provided improvement in the delivery of antioxidants after gastrointestinal digestion. The obtained encapsulated beads can be applied as the natural active ingredient of food, cosmetics, and pharmaceutical products.

## 1. Introduction

According to the standards and requirements of the European Union, there are special legal regulations and procedures related to the procedure of storage and destruction of waste. Because of these reasons, large corporations in the food industry have a growing problem of destruction, recycling, and storage of waste generated during the production process. This phase commonly is more expensive than the production process itself. By constantly increasing the number of people on the planet, the amount of waste also increased representing a serious ecological problem. A significant amount of materials (about 50% of the weight of fresh fruit) are generated due to the processing of citrus fruits. Orange peel is one of the raw materials that is thrown away unused in huge quantities after consumption and processing. It is known that orange peels are rich in bioactive components, such as dietary fiber, pigments, essential oils, antioxidant phenolic compounds [1]. The recovery of these compounds, especially phenolic antioxidants from orange peel waste, is carried out before their final disposition at the landfill. The growing interest of consumers in the use of natural antioxidants caused the development of extraction procedures for the recovery of these compounds from orange peel waste.

The conventional extraction procedures of polyphenolic compounds (maceration, Soxhlet extraction, hydrodistillation) using different concentrations of organic solvents (ethanol, methanol) have been described in the literature [2]. It is known that the yield of phenolic antioxidants increases with the increasing polarity of the solvent [3]. To overcome all disadvantages of conventional extraction techniques (long extraction time and a large volume of solvents), innovative extraction techniques, such as ultrasound-assisted extraction (UAE) [4,5] and supercritical fluid extraction [6] of polyphenolic compounds from orange peels are increasingly used. After the selection of extraction techniques, the next important step is its optimization using appropriate methods [3,7]. Recently, the UAE of phenolic antioxidants has been widely used due to its advantages [4]. The temperature, frequency, power, solvent type, extraction time, and liquid-to-solid ratio are some of the extraction parameters that have an impact on the yield of desired compounds and their functionality. In addition to these parameters, the used apparatus for the UAE also has a significant impact. So far, the ultrasonic processor was used to extract antioxidants from orange peels [4].

The intake of antioxidants through food is important [8], but their use is limited because of chemical instability due to the effect of external factors [9]. In order to provide a sufficient intake of antioxidants, the consumers should be eating a large amount of fruit which can be a serious problem. Thus, the preparation of beads with encapsulated antioxidants would be a useful solution to increase their intake. Microencapsulation is a suitable approach to overcome these problems. Among the many materials used to encapsulate antioxidants, sodium alginate and chitosan are promising agents because of their biocompatibility. Alginate is a biodegradable and non-toxic agent that is commonly used to modify the release of bioactive compounds [10]. Chitosan is also used to produce solid dosage forms with controlled release of the drug substance, and for improving the dissolution of the drug [11]. In recent years, much attention has been paid to the use of combinations of alginate and chitosan in the controlled delivery of antioxidants [12]. Chitosan coats alginate particles making the controlling the diffusion rate of encapsulated compounds, and enabling modification of their structure. The aqueous extract of orange peels was microencapsulated in whey protein concentrate and gum arabic by the coacervation method [13] and multilamellar liposomes [14]. The mixture of *Lepidium sativum* seed gum and *Lepidium perfoliatum* seed gum was used for the encapsulation of essential oil of orange peels in the form of nano-emulsion using an emulsifier Tween 20 [15].

In this paper, the application of central composite design (CCD) and numerical optimization methods was used to optimize the UEA of antioxidants from orange peel performed in an ultrasonic bath. Ethanol (50%, *v*/*v*) was a suitable solvent for the extraction of antioxidants because it is eco-friendly and very effective in this process. The encapsulation of orange peel extract in alginate-chitosan beads was carried out to modify the release of bioactive compounds in the simulated environment of the gastrointestinal tract. The prepared beads were characterized and the type of interactions between them and bioactive compounds was confirmed based on the result of Fourier transform infrared spectroscopy (FTIR). Also, the impact of encapsulation on the antioxidant activity of the extract was estimated.

## 2. Materials and Methods

### 2.1. Reagents

In this study, absolute ethanol (Sani-Hem doo, Novi Becej, Serbia), Folin-Ciocalteu’s reagent (Carlo Erba Reagents, Val de Reuil, France), calcium chloride dihydrate, methanol, acetic acid (Zorka Pharma, Sabac, Serbia), gallic acid (purity 97%) (Merck, Darmstadt, Germany), chlorogenic acid, caffeic acid, coumaric acid, ferulic acid, sinapic acid, 2,2-diphenyl-1-picrylhydrazyl (DPPH), butylhydroxytoluene (BHT) (Sigma Aldrich, St. Louis, MO, USA), sodium salt of alginic acid—very low viscosity (Chem Cruz, Dallas, TX, USA), chitosan molecular weight from 100,000 to 300,000 (Acros Organics, Thermo Fisher Scientific Geel, Belgium) were used. Other used chemicals were p.a. grade (pro analysis).

### 2.2. Plant Material

The sweet oranges (*Citrus × sinensis*) were purchased in the local market. The orange peels were collected in 2020 and then dried at 40 °C for a month. The samples were analyzed in the same year. The moisture content of 13.37% (*w*/*w*) was determined according to the standard procedure. The weight of plant material was measured after drying at 105 °C for 2 h, and then measured every 30 min until the constant weight of the plant material. The obtained value was in accordance with the limits (7–16%) prescribed by the V Yugoslav Pharmacopoeia 2000 [16]. The dried orange peels were ground in an electric mill and sieved on a sieve shaker. A 0.5 mm fraction was chosen for the UAE of antioxidants.

### 2.3. Ultrasound-Assisted Extraction

The UAE of antioxidants was performed by treating orange peels with 50% (*v*/*v*) ethanol, which is the solvent of choice for these bioactive compounds [6]. This ethanol concentration was also obtained in the preliminary studies where the mixture design was applied (these data are not shown in the manuscript) to optimize the solvent for the extraction of antioxidants from orange peels. In that study, the content of methanol, ethanol, and water in the used solvent was analyzed and optimized. An ultrasonic bath (Sonic, Nis, Serbia) with an operating frequency of 40 kHz and ultrasonic power of 150 W was used as an ultrasound source. The liquid extract was separated from the solid matrix by vacuum filtration. All samples were centrifuged on a TH16B centrifuge (Hong Kong, China) at 6000 rpm for 15 min. The extracted sample of 3 mL was dried at 105 °C to a constant sample weight to define its concentration. The prepared liquid extracts were stored in a refrigerator (at 4 °C) until further analysis.

### 2.4. Central Composite Design

The extraction procedure of antioxidants was modeled using central composite design as an experimental design in the response surface methodology. The extraction parameters (extraction time, extraction temperature, and liquid-to-solid ratio) were varied in accordance with a design matrix. Factor levels of defined factors (extraction parameters) are shown in Table 1.

Before modeling, the factor levels were transformed into coded values for better uniformity according to Equation (1):

(1) $$x_{i}=\frac{X_{i}-X_{o}}{\Delta X_{i}}$$

where are, *x_i_*—coded values of factors; *X_i_*—actual values of factors; *X_o_*—actual values of factors in the center point; Δ*X_i_*—step of value change.

The obtained data for total antioxidant content (TAC) were fitted using a second-order polynomial model Equation (2):

(2) $$Y=a_{0}+a_{1}x_{1}+a_{2}x_{2}+a_{3}x_{3}+a_{11}x_{1}^{2}+a_{22}x_{2}^{2}+a_{33}x_{3}^{2}+a_{12}x_{1}x_{2}+a_{13}x_{1}x_{3}+a_{23}x_{2}x_{3}$$

where is, *Y*—the response of the system, *a*_o_—the intercept, *a*_1_, *a*_2_, *a*_3_, *a*_11_, *a*_22_, *a*_33_, *a*_12_, *a*_13_, —the regression coefficients, *x*_1_, *x*_2_, *x*_3_—the factors; *ε*—residue.

### 2.5. Determination of Total Antioxidant Content

The TAC in orange peel extracts was spectrophotometrically determined using Folin-Ciocalteu’s reagent according to the previously described procedure [17]. The sample of extract (0.1 mL) was mixed with 1 mL of Folin-Ciocalteu’s reagent diluted ten times with distilled water, and after a few minutes, 7% (*w*/*w*) sodium carbonate (1 mL) was added. To prepare negative controls, an equivalent amount of distilled water was added instead of sodium carbonate. The TAC was expressed as a gram of gallic acid equivalent per 100 g of dry weight (mg GAE/100 g d.w.). The calibration curve for the determination of gallic acid content was constructed in the concentration range of 0.005–0.300 mg/mL, whose solution series was prepared from the stock solution of 1 mg/mL. The absorbance of the samples was measured at 760 nm in 1 × 1 cm quartz cuvettes on a Varian Cary-100 spectrophotometer (Mulgrave, VIC, Australia) after 90 min.

### 2.6. Phenolic Profile of the Extract

The phenolic profile of the extract was defined using the previously described HPLC method [18]. Separation of the compounds was carried out on the C18 column (Zorbax Eclipse XDB, 4.6 × 250 mm, 5 µm) (Agilent Technologies, Santa Clara, CA, USA) at 20 °C. A 1% aqueous solution of acetic acid and methanol was used as a mobile phase. The injection volume was 20 μL, while the flow rate of the mobile phase was 1 mL/min. The retention time and UV spectrum of the available standards were key in the identification of phenolic compounds. The quantification of compounds was enabled through calibration equations for each standard. The content of identified compounds in the extract was expressed as milligrams per 100 g of dry weight (mg/100 g d.w.).

### 2.7. DPPH Assay

DPPH assay was applied to define the antioxidant activity of the ethanol extract [17]. Briefly, 1 mL of DPPH methanolic solution (3 × 10^−4^ mol/L) was added into 2.5 mL of the diluted solutions of the extract. The incubation time of the samples was 30 min in the dark. The synthetic antioxidant BHT was used as a positive control. The negative control solution was prepared by adding 1 mL of DPPH radicals to 2.5 mL of methanol. Also, the blank samples were prepared by adding 1 mL of methanol to 2.5 mL of the diluted extract solutions. The inhibition of DPPH radicals expressed as a percentage was calculated using Equation (3):

(3) $$I_{DPPH}\left[\%\right]=\frac{A_{C}-(A_{S}-A_{B})}{A_{C}}\times 100$$

where are, *I_DPPH_*—the inhibition of free DPPH radicals, *A_S_*—absorbance of the sample at 517 nm, *A_B_*—absorbance of the blank solution at 517 nm, *A_C_*—the absorbance of the negative control at 517 nm. The half-maximal inhibitory concentration (IC_50_ value) was obtained by interpolation and used to compare the activity of samples with each other.

### 2.8. Encapsulation of Orange Peel Extract in Alginate-Chitosan Microparticles

Alginate-chitosan microparticles with encapsulated orange peel extract were obtained by extrusion method with coaxial airflow. An aqueous solution of alginate (1.5%, *v*/*v*) was first prepared by stirring overnight to completely dissolve it. The ethanol extract was then added to the solution up to the total volume of 66.6 mL. The resulting homogeneous solution was transferred to a plastic syringe of 100 mL with a blunt-tipped metal needle (26 G, 0.45 × 12 mm). The flow rate of the mixture was 33.3 mL/h, while a coaxial airflow pressure was 0.8 bar. The drops were torn off the top of the needle in the form of a jet of small drops, under the action of gravity and coaxial airflow. The droplets were collected in a crosslinking solution with stirring using a magnetic stirrer, causing the droplets to solidify into spherical microparticles. The crosslinking solution consisted of calcium chloride (2%, *w*/*v*) and 0.5% (*w*/*v*) of chitosan. Previously, the chitosan solution was prepared in 0.5% (*v*/*v*) acetic acid that had the role of acidity regulator. The ethanol extract was added to the crosslinking solution until the total volume of 133.3 mL. Instead of the extract, the control alginate-chitosan microparticles have consisted of the equivalent volume of distilled water.

### 2.9. Characterization of Microparticles

#### 2.9.1. Determination of Shape and Size of Alginate-Chitosan Particles

The shape and size of the microparticles were defined by an optical microscope. The roundness of the formed microparticle was estimated based on the sphericity factor (SF) Equation (4) [19]:

(4) $$SF=\frac{D_{max}-D_{per}}{D_{max}+D_{per}}$$

where are, *D_max_*—maximum diameter (mm) of microparticle and *D_per_*—diameter perpendicular to *D_max_* passing through the center of the microparticle (mm). Microparticles are ideal spheres if the SF is around zero. A higher degree of microparticle shape distortion is achieved when the SF has higher values.

#### 2.9.2. Encapsulation Efficiency

The encapsulation efficiency of TAC in microparticles was carried out by their mixing with sodium citrate solution (2%, *w*/*w*) in a ratio of 1:5 (*w*/*w*) for 15 min. The TAC in the citrate solution was determined according to the procedure described in Section 2.5. The encapsulation efficiency of antioxidants represents the ratio of TAC in the citrate solution and initial extract.

#### 2.9.3. FTIR Analysis

The samples were prepared as KBr pastilles and recorded in the wavenumber range from 4000 to 400 cm^−1^ on a spectrophotometer Bomem Hartmann and Braun MB-series (Quebec, QC, Canada).

#### 2.9.4. Determination of Swelling Ability of Alginate-Chitosan Microparticles

The swelling of control and encapsulated microparticles was examined gravimetrically. The simulated gastric fluid (SGF) was the solution of sodium chloride and hydrochloric acid (pH 1.2), while the simulated intestinal fluid (SIF) was phosphate buffer (pH 7.4). The swelling of microparticles was examined at 37 °C, immersing 0.05 g of dry microparticles in the SIF for 2 h. After that, the microparticles were transferred to the SGF for 22 h. Measurements of the weight of swelled microparticles were performed on an analytical balance. The degree of swelling (DS) of microparticles was calculated according to Equation (5):

(5) $$DS\left(\%\right)=\frac{\left(w_{t}-w_{i}\right)}{w_{i}}\times 100$$

where are, *w_t_*—the weight of swelled particles at time *t*, and *w*_0_—the weight of dry particles (xerogel).

#### 2.9.5. In Vitro Release of Antioxidants from Alginate-Chitosan Microparticles

##### In Vitro release

Under SGF and SIF conditions at 37 °C, in vitro release of encapsulated compounds was monitored from dry microparticles. The microparticles (0.6 g) were kept in 30 mL of SGF and 2 mL of aliquots were taken from the medium at certain time intervals for 2 h. After that, exactly 2 mL of fresh SGF was added to the medium. In the collected samples, TAC was determined. The microparticles were further dried and transferred in SIF (30 mL). The release of antioxidants was carried out until the complete degradation of microparticles.

#### 2.9.6. Release Kinetics of Antioxidants from Microparticles

Korsmeyer-Peppas kinetic model was applied to evaluate the kinetics and mechanisms of release of antioxidants from microparticles Equation (6):

(6) $$\frac{m_{t}}{m_{\infty }}=kt^{n}$$

where are, *m_t_* and *m_∞_*—the absolute cumulative amount of antioxidants in time *t* and infinite time, respectively; *k*—characteristic of the alginate-chitosan system; and *n*—the release exponent.

#### 2.9.7. Determination of the Antioxidant Activity of the Extract after Encapsulation

The antioxidant activity was defined in SGF and SIF at 37 °C to the encapsulated microparticles. The sample (0.1 g) was inserted in 10 mL of SGF and SIF, respectively, and an aliquot of 100 μL was taken after 2 h. The antioxidant activity of the obtained samples was determined according to the procedure described in Section 2.7.

### 2.10. Statistical Analysis

The data processing and analysis of variance (ANOVA) of the proposed model were performed in Design Expert 11.0.3.0 (Stat-Ease, Minneapolis, MN, USA). The extractions were repeated three times and the data of TAC were presented as mean ± standard deviation.

## 3. Results

### 3.1. Development of Extraction Procedure of Antioxidants from Orange Peels

The extraction process depends on extraction technique, extraction time, liquid-to-solid ratio, nature of the solvent, the disintegration of plant material, extraction temperature, etc. Optimization is necessary to obtain a quality extract with the least possible consumption of available resources [20]. In this study, the UAE was optimized using the CCD. This approach considers the influence of extraction parameters simultaneously on the defined system response. The one-variable-at-a-time (OVAT) optimization method analyzes the influence of only one parameter on the system response [21]. The disadvantage of this approach is that is impossible to determine the interaction between independent variables (factors). The local optimal conditions are possible to obtain using the OVAT, while the use of CCD leads to the global optimization conditions. The matrix of CCD design with three factors (Table 2) was randomly generated by the software. The total runs were 20 extractions of which 6 extractions were related to the center point. The remaining number of extractions refers to points of full factorial design (2^3^ = 8 extractions) and axial points (3 × 2 = 6 extractions). The minimum TAC (0.86 g GAE/100 g d.w.) was obtained for the extraction of 3 min, the extraction temperature of 50 °C and the liquid-to-solid ratio of 10 mL/g, while the maximum TAC (2.79 g GAE/100 g d.w.) was obtained for the extraction of 30 min, extraction temperature of 60 °C and liquid-to-solid ratio 15 mL/g.

The obtained TAC was fitted with a second-order polynomial model. Equation (7) in the form of coded values, which describes the UAE of antioxidants from orange peels, can be presented as follows:

(7) $$Y=1.93+0.41*A+0.30\times B+0.25*C-2.50\times 10^{-3}*AB+0.11*AC+0.05*BC-0.13*A^{2}-0.08*B^{2}-0.03*C^{2}$$

The terms of a polynomial equation with positive regression coefficients increase the yield of antioxidants in the extract, while those with a negative one reduce the yield. All linear terms of the equation have a positive impact on the defined system response. The influence of extraction parameters on TAC in descending order is following: extraction time > extraction temperature > liquid-to-solid ratio.

The results of ANOVA for the polynomial model at 95% confidence interval are depicted in Table 3. The terms with a *p*-value lower than 0.05 were considered statistically significant. In this model, the interaction between extraction time and the temperature was the only term that was not statistically significant. The lack-of-fit of the model of 0.71 was statistically insignificant so that the model can predict the response well [22].

Statistical parameters of the polynomial model are given in Table 4. The coefficento f determination (*R*^2^) of 0.995 indicated that a 99.5% variation in the TAC could be explained by the proposed model. Adjusted *R*^2^ (0.991) and predicted *R*^2^ (0.997) were in agreement since the difference between them was lower than 0.2. The coefficient of variation (C.V.) should have as soon as possible lower value, and it was lower than 3% in this study. The adequate precision of 55.5 was higher than 4 which indicated a good signal-to-noise ratio.

**Figure 1:**
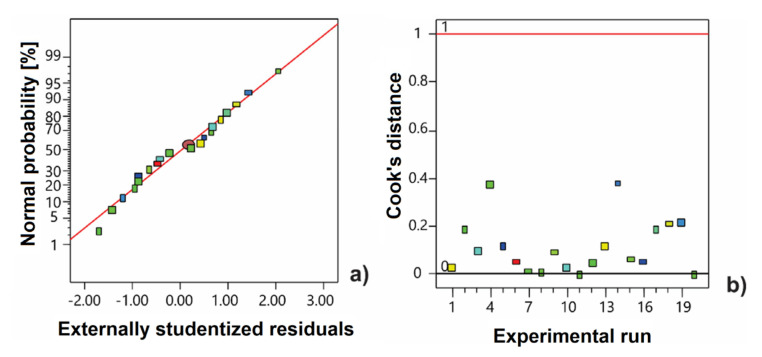
Normal probability plot of residuals (**a**) and Cook’s distance (**b**) for the second-order polynomial model.

The normal probability plot and Cook’s distance are depicted in Figure 1. The residuals had the normal distribution since the data are close to the linear plot (Figure 1a). The model did not have outliers because all distances of data were lower than 1 (Figure 1b).

**Figure 2:**
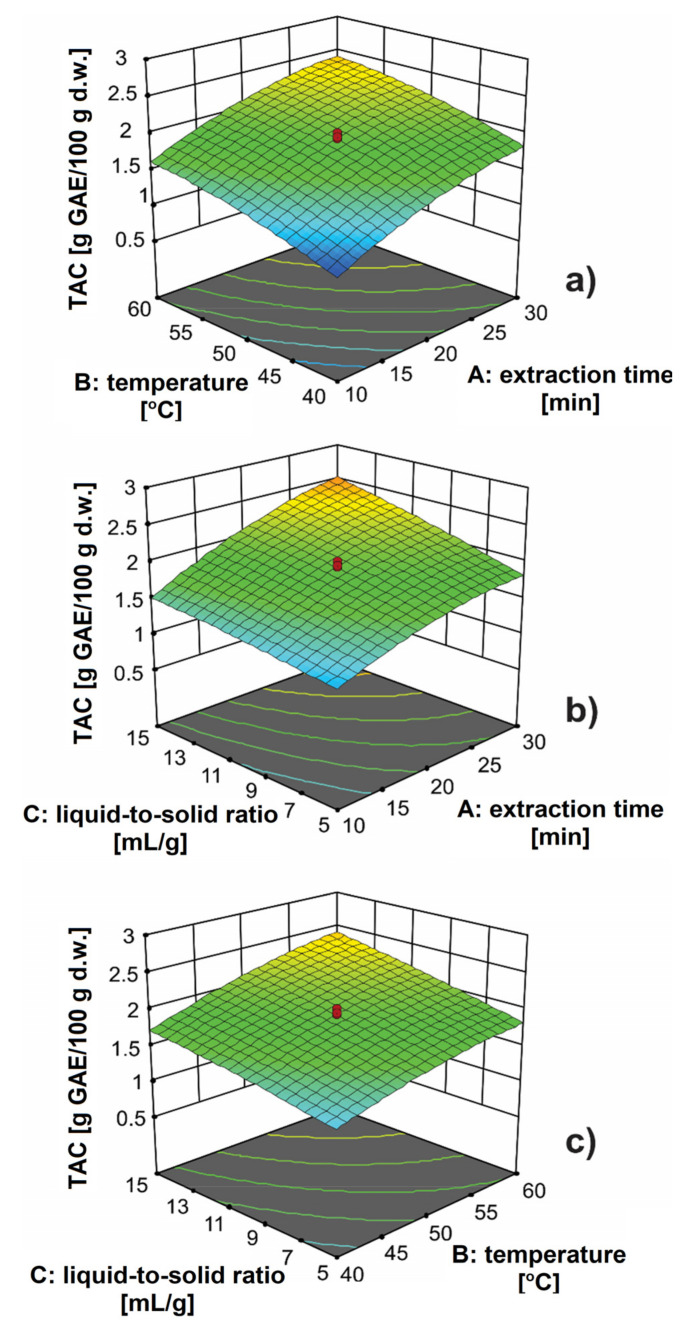
The effect of: (**a**) extraction time and extraction temperature at the liquid-to-solid ratio of 10 mL/g; (**b**) extraction time and the liquid-to-solid ratio at 50 °C; (**c**) extraction temperature and liquid-to-solid ratio for 20 min on the total antioxidant content (TAC).

The 3D plots were constructed to easier estimate the effect of extraction parameters on the defined system response (TAC). In Figure 2a, the interaction between extraction time and extraction temperature at the liquid-to-solid ratio of 10 mL/g is depicted. The response shape did not indicate a strong interaction between the extraction parameters. The TAC was increased with increasing the extraction time. This behavior can be attributed to the fact that with the prolongation of extraction time, there is a better diffusion of antioxidants from the inside of plant cells. Generally, the process has periods of rapid and slow extraction of bioactive compounds [23]. During the period of rapid extraction, the desired bioactive compounds are washed from the surface of the damaged plant cells and dissolved in the solvent. This period is followed by a period of slow extraction, which involves the process of diffusion of bioactive compounds from intact plant cells. A more intense effect of cavitation energy is expressed at the longer extraction times, which causes swelling and wall rupture of cells. In this way, a high rate of diffusion through the cell wall is enabled, which results in the simpler leaching of compounds [24]. With increasing extraction temperature, the TAC was increased due to a decrease in the viscosity of the solvent. This allowed better penetration of the solvent through the plant material. Also, the increase in temperature caused better solubility of antioxidants in the solvent. However, caution should be exercised when increasing the temperature and prolonging the extraction time, because thermodegradation of bioactive compounds can occur. Ma et al. [25] observed a declining trend in hesperidin content from citrus peels due to thermodegradation of antioxidants at higher temperatures and longer extraction times. The positive effect of the extraction time was more pronounced at higher liquid-to-solid ratios (Figure 2b), while at lower values its effect was significantly smaller. With increasing the liquid-to-solid ratio, the TAC was increased, i.e., the concentration gradient was increased during diffusion from the solid into the solvent [7]. In Figure 2c, the interaction between extraction temperature and the liquid-to-solid ratio is presented. The area of higher TAC can be observed for higher liquid-to-solid ratios and higher extraction temperatures. The effect of the extraction temperature was more pronounced at higher liquid-to-solid ratios. Similar behavior was noticed when considering the effect of the liquid-to-solid ratio on the TAC, where the effect of this extraction parameter is more pronounced at higher liquid-to-solid ratios.

The numerical optimization method was used to obtain the optimal conditions for the UAE of antioxidants from orange peels. The optimal conditions were achieved for the extraction time of 30 min, extraction temperature of 60 °C, and the liquid-to-solid ratio of 15 mL/g (Figure 2). Under the given conditions, the UAE was performed to check the validity of the proposed conditions. The experimental and prediction values of TAC were 2.81 and 2.79 g GAE/100 g d.w., respectively. Having in mind this agreement between data, the proposed conditions can be considered adequate. In this way, the predictive ability of the regression model for the designed space is once again confirmed. The development of extraction procedures and the optimization of UAE of antioxidants from orange peels were also studied by several authors. Nakajima et al. [26] analyzed the effect of several solvents (70% methanol, 70% ethanol, 70% ethanol acidified with 1% HCl, 50% ethanol and water) on the yield of extracted antioxidants using the UAE, which was performed at 30 °C for 15 min. The TAC varied between 72.00 mg GAE/mL for aqueous extract and 90.45 mg GAE/mL for 70% (*v*/*v*) ethanol extract acidified with 1% HCl. Barrales et al. [6] also extracted antioxidants from orange peel under the same experimental conditions, but only with 50% (*v*/*v*) ethanol, and obtained the TAC of 5.5 mg GAE/g d.w. Ultrasonic power of 400 W, extraction time of 30 min, and 50% (*v*/*v*) ethanol were proved to be optimal conditions for the UAE of antioxidants from orange peel [4]. Under the given conditions, the TAC in the extract was 105.96 mg GAE/100 g. Therefore, the TAC determined in our study was many times higher than the one obtained for orange peel by other authors. This can be attributed to the difference in the plant material, the extraction technique, and the UAE conditions (ultrasonic bath or extractor with ultrasonic probe).

### 3.2. Characterization of Orange Peel Extract

The TAC of 2.81 g GAE/100 g d.w. in ethanol extract of orange peels was determined by spectrophotometric method. In the literature, the TAC is often misrepresented as the content of total polyphenols. The Folin-Ciocalteu’s reagent reduces the other bioactive compounds in addition to polyphenols [27]. Shehata et al. [28] determined a lower TAC of 345 mg GAE/100 g d.w. in ethanolic extract of red-orange peels prepared by maceration. This can be attributed to the difference in plant material, as well as the extraction technique (ultrasonic bath, extractor with ultrasonic probe, or maceration).

The results of the RP-HPLC method for the orange peel extract are presented in Table 5. Among the identified compounds, there were only phenolic acids. Gallic acid had the highest content (157.08 mg/100 g d.w.), while caffeic acid (127.03 mg/100 g d.w.) was in second place. Ferulic acid had the lowest content (20.91 mg/100 g d.w.). Omoba et al. [29] in the aqueous extract of orange peels determined the content of caffeic acid of 3.5 mg/g. The obtained value was almost 3 times higher than in the extract obtained in this study.

The antioxidant activity of BHT was analyzed in the concentration range of 8–250 µg/mL. Its IC_50_ value of 36.6 µg/mL was determined by interpolation. The concentration of the extract ranged between 8 and 1000 µg/mL, while its IC_50_ value was 65.5 µg/mL. The IC_50_ value of the extract was higher compared to BHT, which indicated that the extract had a slightly lower antioxidant potential than the synthetic antioxidant. Although the antioxidant activity of the extract was lower, it was certainly not negligible. According to the Food and Drug Administration of the United States, ethanol extracts are relatively safe for human consumption compared to other organic solvents [30]. Shehata et al. [28] determined the antioxidant activity of the ethanolic extract of red-orange peel (IC_50_ value of 79.32%) prepared by maceration. The antioxidant activity cannot be compared with the value obtained in our study, since IC_50_ values were expressed in different units.

### 3.3. Characterization Analysis of Microparticles

#### 3.3.1. Microparticle Shape and Size

**Figure 3:**
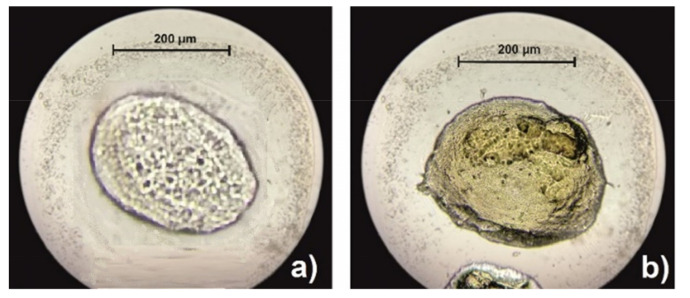
Microscopic image of non-encapsulated (**a**) and encapsulated alginate-chitosan microparticles with orange peel extract (**b**) at a magnification of 10×.

The size and shape of the non-encapsulated and encapsulated alginate-chitosan microparticles are shown in Figure 3. The average sizes of the non-encapsulated and encapsulated alginate-chitosan microparticles were 221 and 252 µm, respectively. The higher values of diameter were measured with encapsulated microparticles, which is most likely due to the higher viscosity of the prepared solution. A polyelectrolyte membrane on the surface of microparticles was occurred due to the electrostatic interaction between alginates and chitosan. By adding alginate to a crosslinking solution, a smaller number of bonds are formed between chitosan and alginate. The reason for this is the competition of Ca^2+^ ions and chitosan for binding to negative sites on the surface of alginate. As a result, a thinner membrane is formed around the microparticle.

The sphericity of non-encapsulated and encapsulated alginate-chitosan microparticles was estimated based on SF values. In this study, the SF was 0.178 which indicates the absence of an ideal spherical morphology. The rough wrinkled surface of the encapsulated microparticles can probably be attributed to the partial collapse of the polymer network during the drying process. The lack of an ideal spherical structure and smoothness has been noted in other studies during the encapsulation of the extract into alginate-chitosan microparticles [31]. The size and sphericity of the microparticle usually depend on the flow rate and the distance between the needle and crosslinking solution [19]. In this study, approximately spherical particles were obtained using a flow rate of 33.3 mL/h and a distance of 15 cm.

#### 3.3.2. Encapsulation Efficiency

Although antioxidants have benefits for human health, their low stability and insolubility are barriers to their use in pharmacy. Antioxidants or phenols are broken down in the extremely acidic environment of gastric juice, which results in low bioavailability. Encapsulation can effectively protect these compounds from the harmful effects of acidic environments. The encapsulation efficiency of orange peel extract in alginate-chitosan microparticles was 89.2%. Given that a rather high value was obtained for the encapsulation efficiency, the proposed extrusion method with coaxial airflow was effective for the encapsulation of ethanolic orange peel extract. The relatively small amount of non-encapsulated extract was most likely due to losses during the encapsulation or rapid degradation of some very unstable compounds. Approximately the same encapsulation efficiency was determined during the encapsulation of phenolic compounds extracted from waste wine (about 80%) [32].

#### 3.3.3. FTIR Analysis

**Figure 4:**
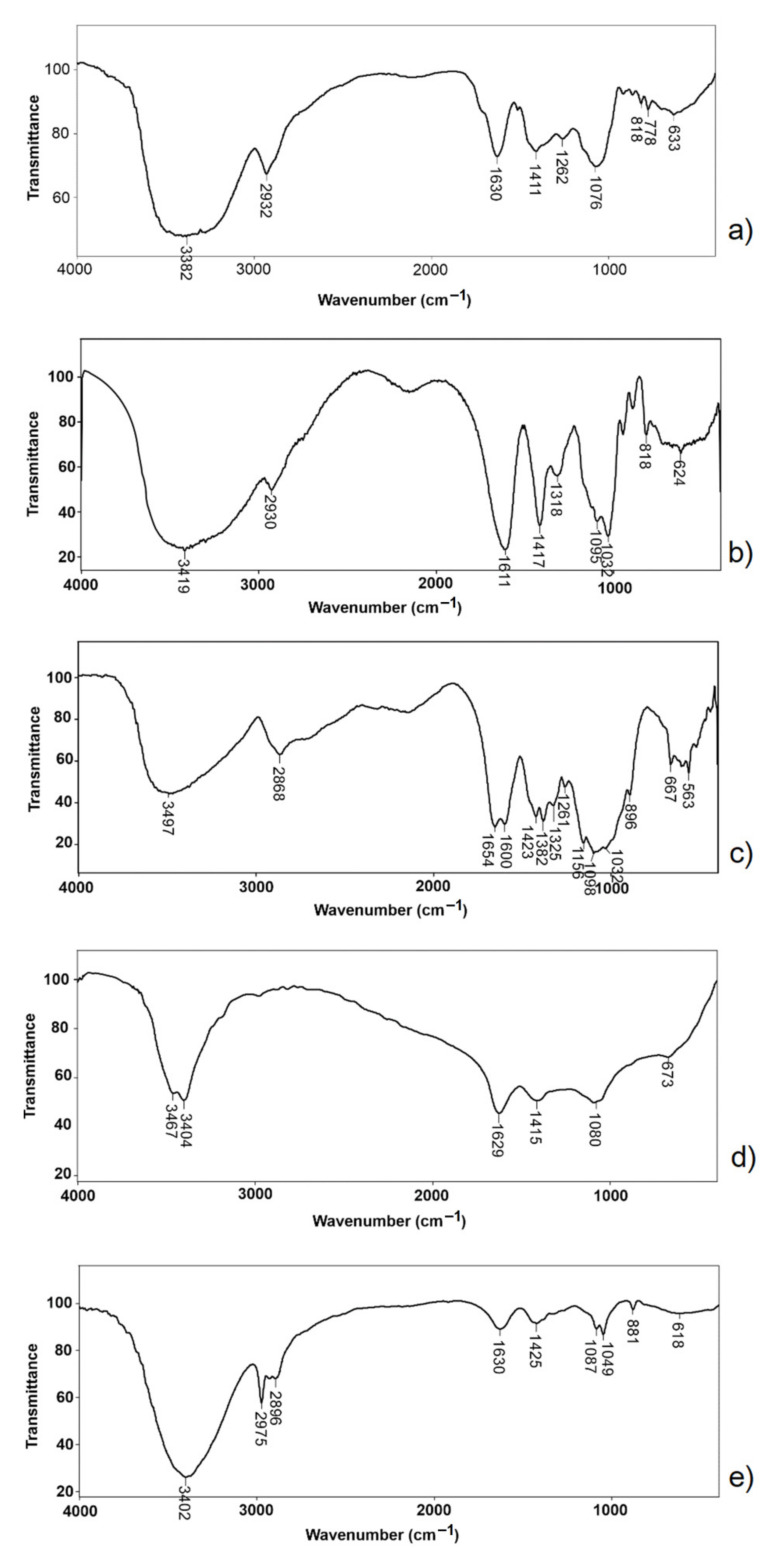
FTIR spectra of orange peel extract (**a**), sodium alginate (**b**), chitosan (**c**), non-encapsulated (**d**), and encapsulated (**e**) alginate-chitosan microparticles.

Functional groups and the appearance of intermolecular interactions in the microparticle were examined using the FTIR method [33]. The FTIR spectra of orange peel extract, sodium alginate, chitosan, non-encapsulated, and encapsulated alginate-chitosan microparticles are shown in Figure 4. The FTIR spectrum of orange peel extract (Figure 4a) had a wide band at 3382 cm^−1^ corresponding to the valence of O-H vibrations. The small band at 2932 cm^−1^ originated from the valence vibration of the sp^3^ C-H bond. The band at 1630 cm^−1^ originated from the valence vibration of the C=O bond, while the band at 1411 cm^−1^ corresponded to the deformation vibrations of the C–C bond in phenolic groups. The absorption band at 1076 cm^−1^ was a consequence of the valence vibration of C-O phenolic bonds. In the spectrum of orange peel extract, the bands in the range of 818–633 cm^−1^ were identified and corresponded to the vibration of the aromatic ring. The spectrum of sodium alginate (Figure 4b) showed the characteristic bands at 3419 cm^−1^ (the valence vibration of OH groups), 2930 cm^−1^ (the valence vibration of sp^3^ CH bond), 1611 cm^−1,^ and 1417 cm^−1^ (asymmetric and symmetric valence vibration of COO^−^ group) and 1032 cm^−1^ (the valence vibration of C-O-C). Similar absorption bands were observed by Khorshidian et al. [31]. As shown in Figure 4c, the characteristic chitosan bands were at 3497 cm^−1^ (the valence vibration of O-H groups), 2868 cm^−1^ (the valence vibration of C-H bonds), 1654 cm^−1^ (the valence vibration of C=O groups), amide I representing the structure of *N*-acetylglucosamine), 1600 cm^−1^ (the valence vibration of N-H bonds representing *N*-acetylated residues), 1423 cm^−1^ (the valence vibration of N-H bonds, amide II representing glucosamine functional groups), 1382 cm^−1^ (the valence vibration of N-H bonds, amide III), 1156 cm^−1^ (the valence vibration of C-O-C bonds) and 896 cm^−1^ (pyranose ring) [31]. Comparing the spectra of non-encapsulated microparticles (Figure 4d) with sodium alginate (b) and chitosan (c), it was found that most of the specific bands of the alginate and chitosan were present in the spectrum of microparticles with some slight shifts. The valence vibration bands of the O-H bonds at 3419 cm^−1^ (alginate) and 3497 cm^−1^ (chitosan) were shifted to lower wavenumbers of 3467 cm^−1^ and 3404 cm^−1^, respectively. The formation of polyelectrolyte complex was confirmed between alginate and chitosan based on the decrease in band intensity at 1629 cm^−1^ (COO¯ group of alginate) and the disappearance of band at 1600 cm^−1^ (amino group of chitosan) [31]. Some bands that occurred in the chitosan spectrum were not observed in the spectrum of non-encapsulated microparticles. The reason for this was the presence of multiple polymer interactions, such as electrostatic and hydrogen bonding. The spectrum of encapsulated microparticles (Figure 4e) was a combination of the spectra of extract and non-encapsulated microparticles. The appearance of some bands at 1087, 1049, and 618 cm^−1^ in the spectrum of encapsulated microparticles (Figure 4e) was a result of the encapsulation of the extract in the alginate-chitosan matrix.

#### 3.3.4. Swelling Study

**Figure 5:**
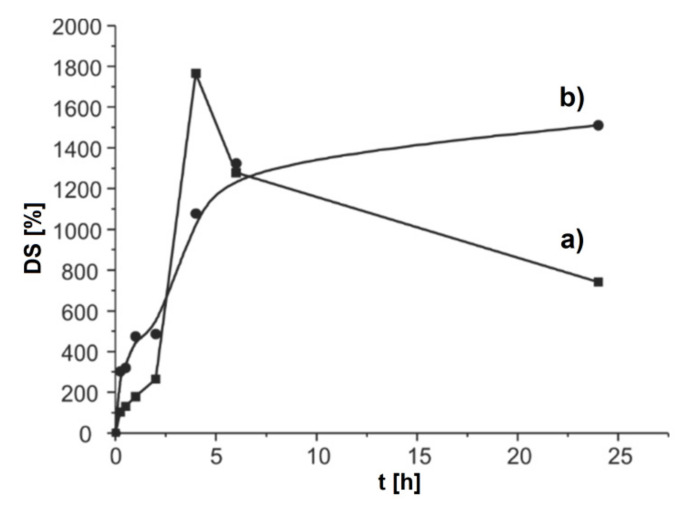
The dependency between the DS of alginate-chitosan microparticles and time in solutions of different pH values: (**a**) simulated gastric fluid (SGF), and (**b**) simulated intestinal fluid (SIF) at 37 °C.

Microparticle swelling was monitored in the SGF for 2 h. The microparticles were then filtered and transferred to the SIF, where the swelling was monitored for a further 22 h. All measurements were performed at 37 °C. The impact of time and pH value of the used medium on the swelling of non-encapsulated and encapsulated alginate-chitosan microparticles is shown in Figure 5.

At pH 1.2, the DS of non-encapsulated microparticles was increased intensively and reached a maximum value of 265.0% after 2 h. At the same pH, the DS of encapsulated microparticles was higher and the maximum value of 265.0% was reached after 2 h. Hydration of hydrophilic groups of alginate and chitosan was the cause of the increase in the DS in both types of microparticles. Also, the protonation of the amino groups of chitosan was another key factor affecting the swelling at low pH values. In that case, repulsive forces are formed, which affect the local separation of the two polymers and the creation of pores that enable easier water penetration. Higher DS of encapsulated microparticles was expected since there was hydration of hydrophilic groups in the extract. When the microparticles were transferred to the SIF (pH 7.4), they swelled faster and achieved higher DS. In the non-encapsulated microparticles, the DS was being grown up to 4 h and reached a maximum value of 1765.4%. After that, the DS began to slowly decline by 24 h. The reason for the decrease in the DS was the dissolution of alginate in the hydrated microparticle. In the end, the microparticles were completely dissolved. Unlike non-encapsulated microparticles, the DS in encapsulated microparticles was constantly increased for up to 24 h. This is most likely a consequence of the ionization of carboxyl groups and the rupture of hydrogen bonds, which leads to the expansion of the polymer network, and thus to the increase in the swelling capacity of the particles. Based on the results obtained in this study, the swelling ability of microparticles depended on the pH value of the surrounding medium. With increasing pH value, the ability to absorb water from the surrounding medium was also increased. Similar behavior of the DS of alginate-chitosan microparticles was explained by Pasparakis and Bouropoulos [34]. pH-dependent swelling of alginate-chitosan microparticles can be attributed to the pH sensitivity of the alginate-chitosan polyelectrolyte complex. This complex was based on the electrostatic attraction of two oppositely charged ions, alginate as a polyanion (weak acid) and chitosan as a polycation (a weak base). At lower pH values (artificial gastric juice), –COO^−^ ions of alginate chains were converted into their protonated –COOH form. That resulted in the decrease in intensity of attractive forces between alginates and chitosans, despite the high degree of protonation of -NH_2_ groups of chitosans, which existed in the form of –NH_3_^+^ ions. With increasing the pH value of the surrounding medium, the number of protonated –NH_2_ groups of chitosan was decreased, but at the same time, the number of –COO^−^ ions in alginate chains was increased. In this way, the conditions were created for stronger and closer bonding of two polymers, i.e., there was an increase in the stability of microparticles, and thus a decrease in the ability to absorb water.

#### 3.3.5. In Vitro Study of Antioxidant Release in Simulated Gastrointestinal Fluids

**Figure 6:**
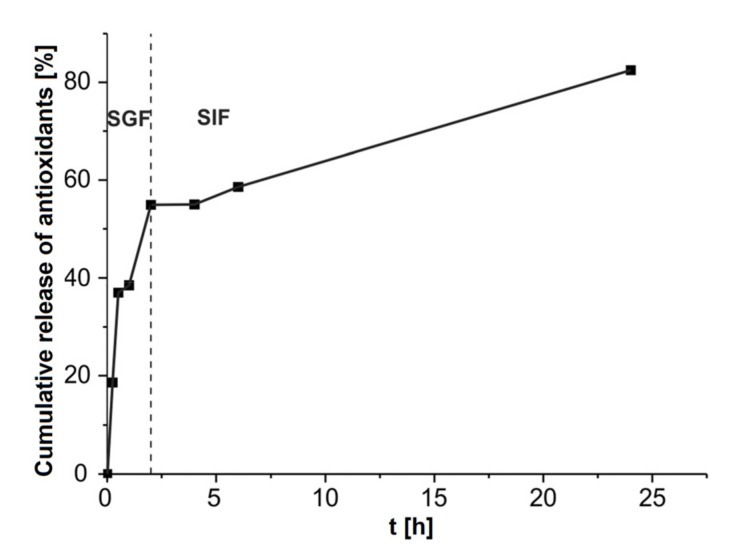
Release of orange peel antioxidants from alginate-chitosan microparticles in the simulated gastric fluid (SGF) and simulated intestinal fluid (SIF).

In order to prolong the release time of antioxidants, the orange peel extract was encapsulated in alginate-chitosan microparticles. In Figure 6, the release of antioxidants from the microparticles in the SGF and SIF are depicted. Under the SGF conditions, there was a rapid initial release of antioxidants from microparticles (18.6%) during the first 15 min. This result was expected due to the large gradient of antioxidant concentrations between the interior of the microparticles and the surrounding medium. It is known that the rapid release of antioxidants from alginate particles occurs in the first 10 min. The release time of antioxidants from alginate-chitosan microparticles was slightly extended compared to alginate particles with the encapsulated extract. This result indicated that chitosan loaded the alginate particle, thus forming an additional layer that prevented the diffusion of antioxidants from the inside of the matrix into the surrounding medium. The percentage of antioxidants released from microparticles was 55% up to 2 h, followed by a period of a slower release. In the SIF, 82.5% of antioxidants were released within 24 h.

In order to predict the correlation of antioxidant release from alginate-chitosan microparticles in vitro, the obtained results for cumulative antioxidant release were modeled by the Korsmeyer-Peppas model. The kinetic parameters of the antioxidant release profile from microparticles were: R^2^ of 0.835 and diffusion exponent *n* = 0.32. The calculated value of the diffusion exponent indicates that the release of antioxidants from microparticles obeyed simple Fick diffusion since the value was lower than 0.5 [35].

#### 3.3.6. Antioxidant Activity of Encapsulated Alginate-Chitosan Microparticles

The antioxidant activity of the extract was determined after 2 h of antioxidant release in the SGF and SIF to estimate the effect of the encapsulation process. The IC_50_ values of 2.2 μg/mL in SGF and 8.57 μg/mL in SIF indicated the antioxidant activity of the extract was higher in SGF. The antioxidant activity of the extract was increased after encapsulation because the IC_50_ value of the extract (65.5 μg/mL) before encapsulation had the highest value compared with the other samples. This increase in antioxidant activity can be attributed to the antioxidant activity of alginates [36] and chitosan [37].

## 4. Conclusions

Using CCD and the numerical optimization method, the UAE of antioxidants from orange peels were optimized. Among phenolic compounds, gallic acid had the highest content (157.08 mg/100 g d.w.), while ferulic acid had the lowest content (20.91 mg/100 g d.w.). The IC_50_ value of 65.5 µg/mL for the extract was defined based on the DPPH assay. The extrusion method with coaxial airflow was used to prepare alginate-chitosan microparticles in which ethanol extract of orange peels was encapsulated. The encapsulation improved the release of antioxidants and preserved the antioxidant activity of orange peel extract in the gastrointestinal tract. The orange peel extract enriched with antioxidants and encapsulated in the alginate-chitosan microparticles has proven safe for pharmaceutical, cosmetic, and nutritional purposes. This is supported by the fact that no toxic solvents were used during the extraction of antioxidants.

## Figures and Tables

**Table 1:** Factor levels of extraction parameters.

Factor	Coded Values				
	−α	−1	0	+1	+α
	Actual Values				
extraction time [min]	3	10	20	30	37
extraction temperature [°C]	33	40	50	60	67
liquid-to-solid ratio [mL/g]	2	5	10	15	18

**Table 2:** CCD matrix with three factors and content of total antioxidants.

Std.	Exp. Run	Factor 1	Factor 2	Factor 3	Response Y: TAC [g GAE/100 g d.w.]
		A: Extraction Time [min]	B: Temperature [°C]	C: Liquid-to-Solid Ratio [mL/g]	
14	1	20	50	18	2.28 ± 0.06
7	2	10	60	15	1.76 ± 0.05
3	3	10	60	5	1.41 ± 0.08
4	4	30	60	5	1.95 ± 0.08
9	5	3	50	10	0.86 ± 0.04
8	6	30	60	15	2.79 ± 0.09
17	7 *	20	50	10	1.89 ± 0.08
19	8 *	20	50	10	1.96 ± 0.04
6	9	30	40	15	2.08 ± 0.02
13	10	20	50	2	1.41 ± 0.09
15	11 *	20	50	10	1.92 ± 0.05
20	12 *	20	50	10	1.86 ± 0.10
10	13	37	50	10	2.28 ± 0.12
5	14	10	40	15	1.11 ± 0.09
16	15 *	20	50	10	2.01 ± 0.11
1	16	10	40	5	0.90 ± 0.09
2	17	30	40	5	1.52 ± 0.11
12	18	20	67	10	2.24 ± 0.12
11	19	20	33	10	1.15 ± 0.06
18	20 *	20	50	10	1.94 ± 0.07

Std—standard order, TAC—total antioxidant content, *—the center point of the design. The values are presented as means ± SD (*n* = 3).

**Table 3:** ANOVA test for the second-order polynomial model.

	Sum of Squares	Df	Middle Square	*F*-Value	*p*-Value
Model	4.780	9	0.531	221.8	2.59 × 10^−10^
A-time	2.254	1	2.254	941.4	3.17 × 10^−11^
B-temperature	1.251	1	1.251	522.4	5.80 × 10^−10^
C-liquid-to-solid ratio	0.858	1	0.858	358.4	3.67 × 10^−9^
AB	0.000	1	0.000	2.1 × 10^−2^	0.888
AC	0.088	1	0.088	36.8	1.20 × 10^−4^
BC	0.022	1	0.022	9.2	1.26 × 10^−2^
A²	0.233	1	0.233	97.4	1.80 × 10^−6^
B²	0.099	1	0.099	41.5	7.45 × 10^−5^
C²	0.013	1	0.013	5.4	4.24 × 10^−2^
Residue	0.024	10	0.002		
Lack of fit	0.010	5	0.002	0.7	0.642
Pure error	0.014	5	0.003		
Total correction	4.804	19			

**A**—extraction time; **B**—extraction temperature; **C**—liquid-to-solid ratio; **df**—degree of freedom.

**Table 4:** Fitting statistics for the polynomial model.

Std. dev.	0.05	R²	0.995
Mean value	1.77	Adj. R²	0.991
C.V. [%]	2.77	Pred. R²	0.979
		Adequate Precision	55.5

Std. dev.—standard deviation; C.V.—coefficient of variation; R^2^—coefficient of determination.

**Table 5:** Content of phenolic acids in ethanolic extract of orange peels.

Phenolic Acid	λ [nm]	t_R_ [min]	Calibration Curve	Concentration [mg/100 g d.w.]
gallic acid	278	5.38	A = 1.320 × 10^−5^ C + 0.287 (*R*^2^ = 0.9995)	157.08 ± 1.26
chlorogenic acid	300	29.12	A = 1.601 × 10^−5^ C + 1.447 (*R*^2^ = 0.9987)	29.60 ± 0.59
caffeic acid	300	31.02	A = 8.406 × 10^−6^ C + 0.209 (*R*^2^ = 0.9982)	127.03 ± 1.32
coumaric acid	300	45.98	A = 5.706 × 10^−6^ C + 1.241 (*R*^2^ = 0.9991)	26.15 ± 0.89
ferulic acid	300	50.09	A = 9.320 × 10^−6^ C − 0.101 (*R*^2^ = 0.9989)	20.91 ± 0.28
sinapic acid	300	51.12	A = 1.120 × 10^−5^ C − 0.242 (*R*^2^ = 0.9993)	28.61 ± 0.98

The values are presented as means ± SD (*n* = 3). A—peak area; C—the concentration of a standard solution (μg mL^−1^); *R*^2^—coefficient of determination.

## Data Availability

The data is contained within the article.
